# **Assessment of the cardiac vagal reflex**: **validation of the 4-s exercise test during simulated wheelchair propulsion**

**DOI:** 10.1007/s00421-026-06147-3

**Published:** 2026-02-07

**Authors:** Gustavo C. Bezerra, Marcela S. Araújo, Frederico Ribeiro Neto, Lauro C. Vianna

**Affiliations:** 1 NeuroV̇ASQ̇ - Integrative Physiology Laboratory, Faculty of Physical Education, University of Brasília, Darcy Ribeiro Campus, Brasília, DF 70910-900 Brazil; 2 Paralympic Sports Program, SARAH Rehabilitation Hospital Network, Brasília, DF Brazil

**Keywords:** Parasympathetic nervous system, Autonomic assessment, Cardiac vagal withdrawal, RR interval

## Abstract

**Purpose:**

The rapid heart rate increase during the first seconds of exercise is primarily mediated by cardiac vagal withdrawal, which can be assessed by the 4-s exercise test (4sET), a pharmacologically validated and highly reliable procedure. However, the need for a cycle ergometer limits its applicability. Thus, we aimed to test the validity of a modified 4sET using wheelchair propulsion simulation (WPS) by comparing it with the traditional cycle ergometer protocol.

**Methods:**

Sixty healthy adults (30 men, 30 women; 22 ± 2 years) performed, in randomized order, three repetitions each of the traditional (LEG 4sET) and modified (WPS) protocols. RR intervals were recorded via electrocardiography, and the cardiac vagal index (CVI) was calculated as the ratio of the last pre-exercise RR interval (RRB) to the shortest exercise RR interval (RRC).

**Results:**

Mean CVI was lower for WPS compared with LEG (1.42 ± 0.03 vs. 1.48 ± 0.03; P = 0.001). Based on identity plots, a correction equation was derived for WPS values < 1.50: y = 0.7706x + 0.3861 (r^2^ = 0.63; P < 0.001). After correction, CVI did not differ between protocols (1.48 ± 0.17 vs. 1.48 ± 0.20; P = 0.854). Furthermore, a high and significant intraclass correlation coefficient (ICC) was found for the CVI between the protocols (ICC = 0.87; [0.74–0.93]; P < 0.05), and Bland–Altman analysis showed negligible bias and acceptable limits of agreement for the corrected WPS CVI.

**Conclusion:**

The WPS shows strong agreement with the traditional cycle ergometer 4sET, supporting its validity as a simpler and more accessible method for assessing dynamic cardiac vagal control.

## Introduction

The autonomic nervous system, through its sympathetic and parasympathetic branches, plays a crucial role in cardiovascular regulation during exercise (White and Raven 2014; Teixeira et al. 2020). At the very onset of dynamic activity, heart rate rises abruptly within the first few seconds, driven primarily by cardiac vagal withdrawal mediated by central command and mechanically sensitive afferents of the exercise pressor reflex (Fisher et al. 2015; Teixeira and Vianna 2022; Lehnen et al. 2025). More importantly, because impaired vagal function is strongly linked to adverse cardiovascular outcomes (Thayer and Lane 2007; Thayer et al. 2010; Goldberger et al. 2019), methods capable of accurately and practically assessing parasympathetic tone, especially those that capture the rapid vagal withdrawal at exercise onset, may offer valuable opportunities for early detection of autonomic dysfunction and for informing preventive or therapeutic strategies.

The 4-s exercise test (4sET) is a pharmacologically validated, non-invasive procedure specifically designed to assess cardiac vagal withdrawal during the transition from rest to exercise, with minimal sympathetic influence (Araújo et al. 1992). The protocol involves 4 s of rapid, unloaded cycling initiated at the 4th second of a 12-s maximal inspiratory apnea, and yields the cardiac vagal index (CVI), defined as the ratio between the longest pre-exercise RR interval and the shortest RR interval during exercise. The 4sET has demonstrated high reproducibility (Araújo et al. 2003), operational simplicity (Paiva et al. 2011), and broad applicability across diverse populations, including patients with coronary artery disease (Ricardo et al. 2005), children with asthma (Knopfli et al. 2005), obese adolescents (Oliveira et al. 2019), older adults (Millar et al. 2009), and endurance athletes (Zaniqueli et al. 2014).

Despite these advantages, the requirement for a cycle ergometer limits the use of the 4sET in individuals with severe mobility impairments, such as wheelchair users or those with high-level spinal cord injury, whose exercise-induced cardiac vagal responses remain poorly characterized. Notably, Silva et al. (2008) demonstrated similar cardiac vagal withdrawal during arm and leg versions of the 4sET performed on a cycle ergometer, suggesting that alternative movement patterns may yield comparable results. This finding opens the possibility of adapting the 4sET to simulate wheelchair propulsion, offering a valid and operationally simpler approach for populations with lower-limb or global motor impairments. Therefore, the present study aimed to validate a modified 4sET protocol using wheelchair propulsion simulation (WPS 4sET) by comparing it to the traditional lower-limb cycling protocol (LEG 4sET). We hypothesized that the WPS 4sET would elicit a cardiac vagal withdrawal comparable to that observed in the LEG 4sET.

## Methods

## Ethical approval

The study was approved by the local institutional research committee (CAAE: 81526824.3.0000.0030). All subjects participated in the present study voluntarily, receiving no financial incentive. Participants read and signed a specific informed consent form before data collection and all experimental procedures and protocols conformed to the standards set by the Declaration of Helsinki of the World Medical Association Ethics Code. All data are only available upon request to the senior author (legal guardian of the data). This option follows the local laws and ethical regulations that apply to confidentiality of clinical/medical data.

### Sample

A total of sixty healthy volunteers (30 men and 30 women) with a mean (± SD) age of 22 ± 2 years, body weight of 70 ± 15 kg and height of 171 ± 10 cm were enrolled in this study. Participants reported no history of heart disease and no use of medications known to affect autonomic function. All subjects reported to be recreationally active, engaging in regular physical activity for at least 6 consecutive months with a minimum frequency of 3 days per week in sessions lasting ≥ 30 min. Recruitment was conducted through social media posts and flyers distributed throughout the university. Prior to the experimental session, participants were instructed to abstain from caffeinated beverages and strenuous physical activity for 12 h, alcohol for at least 24 h, and to arrive at the laboratory at least 2 h postprandial.

### Measurements

**Fig. 1:**
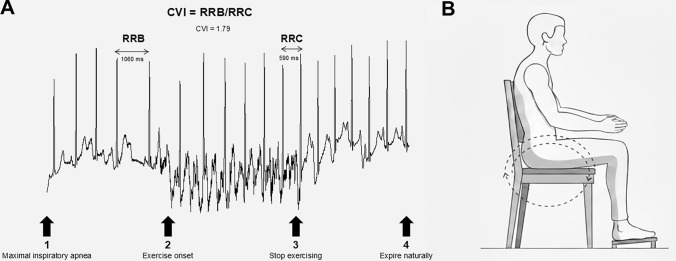
Schematic representation of the cardiac vagal index (CVI) calculation and the wheelchair propulsion simulation (WPS) during the 4-s exercise test (4sET). **A** Calculation of the CVI as the ratio between the longest RR interval immediately before exercise onset (RRB) and the shortest RR interval during the first 4 s of movement (RRC). **B** Wheelchair propulsion simulation (WPS). Participants are seated in a standard chair with back support and both feet flat on the floor. The movement consists of a coordinated upper-limb action initiated by slight elbow flexion combined with shoulder extension, followed by shoulder flexion and elbow extension, simulating the propulsion phase of wheelchair movement

The 4-s exercise test (4sET) was used to assess cardiac vagal activity at the onset of dynamic exercise. Briefly, the 4sET consists of cycling as fast as possible on an unloaded cycle ergometer (Inbramed, CG-04, Brazil) from the 4th to the 8th second of a maximum inspiratory apnea of 12 s. The stages of the maneuver are characterized by four verbal commands, which are: 1) perform a maximal inspiratory apnea, primarily through the mouth; 2) cycle as fast as possible; 3) stop cycling; and 4) expire naturally. Ag–AgCl electrodes were placed on the fifth intercostal space of the anterior axillary line, one on each side, and another on the forehead, totaling 3 electrodes. This lead was used to reduce artifacts in the ECG tracings, mainly in the arm procedure (Silva et al. 2008). To quantify cardiac vagal index (CVI), the duration of two RR intervals was measured: the longest RR interval duration obtained immediately before the onset of exercise or the first one after the onset (RRB) and the shortest RR interval duration during exercise (RRC). The ratio between RRB and RRC provides the CVI, a dimensionless variable, considered to reflect the cardiac vagal withdrawal at the onset of exercise (Fig. 1A). An elastic respiratory band placed in a stable position around the abdomen to monitor respiratory movements and confirm the onset, maintenance, and end of the inspiratory apnea during the 4-s exercise test. All data were collected at a 1,000 Hz sampling rate and stored for offline analysis (PowerLab 16/35, software LabChart 8; AD Instruments, Australia).

### Study protocol

Each participant performed the 4sET using two different approaches, administered in random order. In the LEG 4sET protocol, the seat height of the cycle ergometer was adjusted to allow a slight knee flexion when the pedal reached its lowest point. In the WPS 4sET protocol, participants were seated in a standard chair with back support and both feet flat on the floor, and no equipment was required. The wheelchair propulsion simulation consisted of initiating the movement with a slight elbow flexion at the beginning of shoulder extension and completing it with shoulder flexion followed by elbow extension (Fig. 1B). This adaptation eliminated the need for a cycle ergometer while preserving the dynamic effort pattern of the original protocol. For both protocols, participants were instructed to perform the movement as fast and forcefully as possible while maintaining trunk stability and avoiding unnecessary muscle contractions. Prior to testing, a 5–10 min rest period was provided to ensure stabilization of heart rate. After baseline measurements, the 4sET was performed six times in total—three repetitions for each protocol. Between trials, RR intervals were allowed to return to baseline, which typically required 1–2 min. For analysis, the highest cardiac vagal index (CVI) value obtained from each protocol was used. All tests were conducted in a temperature-controlled room (24 °C) with minimal external stimuli.

### Statistical analysis

The Kolmogorov–Smirnov test was used to assess the normality of data distribution. All variables were normally distributed except for the WPS 4sET CVI. For each protocol, the highest cardiac vagal index (CVI) value from the three repetitions was selected for analysis, in accordance with previous 4sET studies, to minimize intra-individual variability and maximize the stability of the measure. Reliability between protocols was assessed using the intraclass correlation coefficient (ICC; two-way mixed effects, average measures, absolute agreement). ICC values were interpreted qualitatively as follows: < 0.39, poor; 0.40–0.59, moderate/fair; 0.60–0.74, good; 0.75–0.89, very good; and > 0.90, excellent (Dillon et al. 2020). The typical error of measurement (TEM; within-subject standard error of measurement) was also calculated as an additional index of reliability (Hopkins 2000) and expressed as a percentage. Agreement between LEG and WPS 4sET CVI was further examined using both linear regression model and Bland–Altman analysis (Bland and Altman 1986). For the Bland–Altman plot, the difference between LEG and WPS 4sET CVI values was plotted against their mean, and the mean bias and limits of agreement (mean difference ± 1.96 standard deviation) were calculated to assess systematic bias and agreement between protocols. A 1-sample *t* test was used to help assess if the mean difference between the 2 measurement protocols was significantly different from zero. Differences between variables were tested using a two-way repeated-measures ANOVA. Results are presented as mean ± standard error of the mean (SEM), with reproducibility indices reported as mean values and 95% confidence intervals (CI). The significance level was set at 5%, and all tests were two-tailed. All statistical analyses were performed with the software IBM SPSS Statistics (version 20; SPSS, USA) for Windows and Prism (version 8.01; GraphPad, USA) was utilized to generate figures.

## Results

**Fig. 2:**
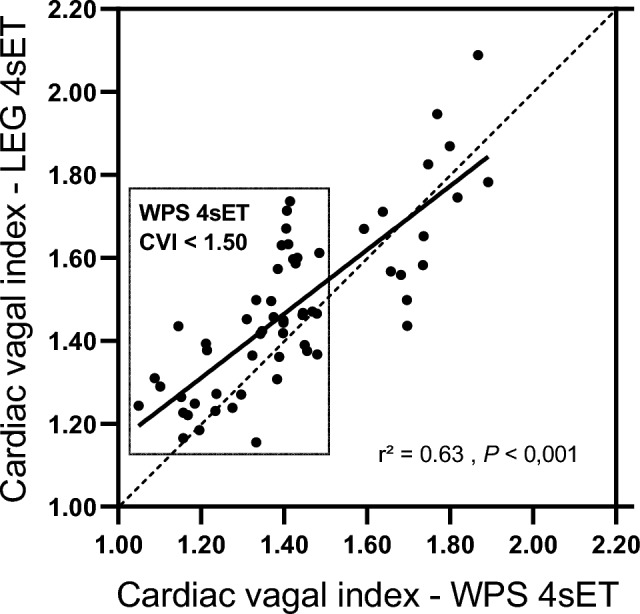
Relationship between the cardiac vagal index (CVI) of LEG and WPS 4-s exercise test (4sET). The regression equation is: *y* = 0.7706x + 0.3861 (*r*^*2*^ = 0.634, *SEE* = 0.12, *P* < 0.001, *n* = 60). The thick line indicates the regression line. The dashed line indicates the line of identity

Table 1 summarizes the RRB and RRC values for the LEG and WPS 4sET protocols. Mean CVI was significantly lower in the WPS compared with the LEG 4sET (1.42 ± 0.03 vs. 1.48 ± 0.03; TEM = 6.4%; P = 0.001). The Bland–Altman analysis revealed a bias of 0.06, with 95% limits of agreement ranging from -0.197 to 0.317, and the mean difference between WPS and LEG 4sET CVI values was significantly different from zero (t = 3.5; P = 0.008). Despite this difference, reliability between protocols was very good, as indicated by a high and significant ICC (ri = 0.868; 95% CI = 0.744–0.928; P < 0.05). Figure 2 shows the relationship between CVI values obtained in WPS and LEG 4sET, demonstrating a significant coefficient of determination (r^2^ = 0.63, P < 0.001) and a regression line closely aligned with the line of identity. The standard error of estimate (SEE) was calculated to indicate the accuracy of the regression equation.

**Table 1:** Comparison between LEG and WPS 4sET

	LEG 4sET	WPS 4sET	*P-*value	*ri* (CI 95%)
RRB (ms)	989 ± 19	923 ± 21	0.001	0.89* (0.53–0.96)
RRC (ms)	673 ± 12	653 ± 11	0.010	0.88* (0.79–0.93)
CVI	1.48 ± 0.03	1.42 ± 0.03	0.001	0.87* (0.74–0.93)

Values are means ± SEM of 60 subjects. *RRB* RR interval duration obtained immediately before, or the first one, after the exercise onset; *RRC* shortest RR interval duration during exercise; *CVI* cardiac vagal index, ratio between RRB and RRC; *ri* intraclass correlation coefficient. Significant differences between LEG and WPS 4sET are denoted. *P < 0.05

Visual inspection of the identity plot revealed that, for WPS 4sET CVI values < 1.50, the corresponding LEG 4sET values tended to be higher. To address this bias and improve agreement, we derived the following correction equation:

$$y = 0.7706x + 0.3861\,\,\left( {r^{2} = 0.634,\,SEE = 0.12,\,P < 0.001,\,n = 60} \right)$$

**Fig. 3:**
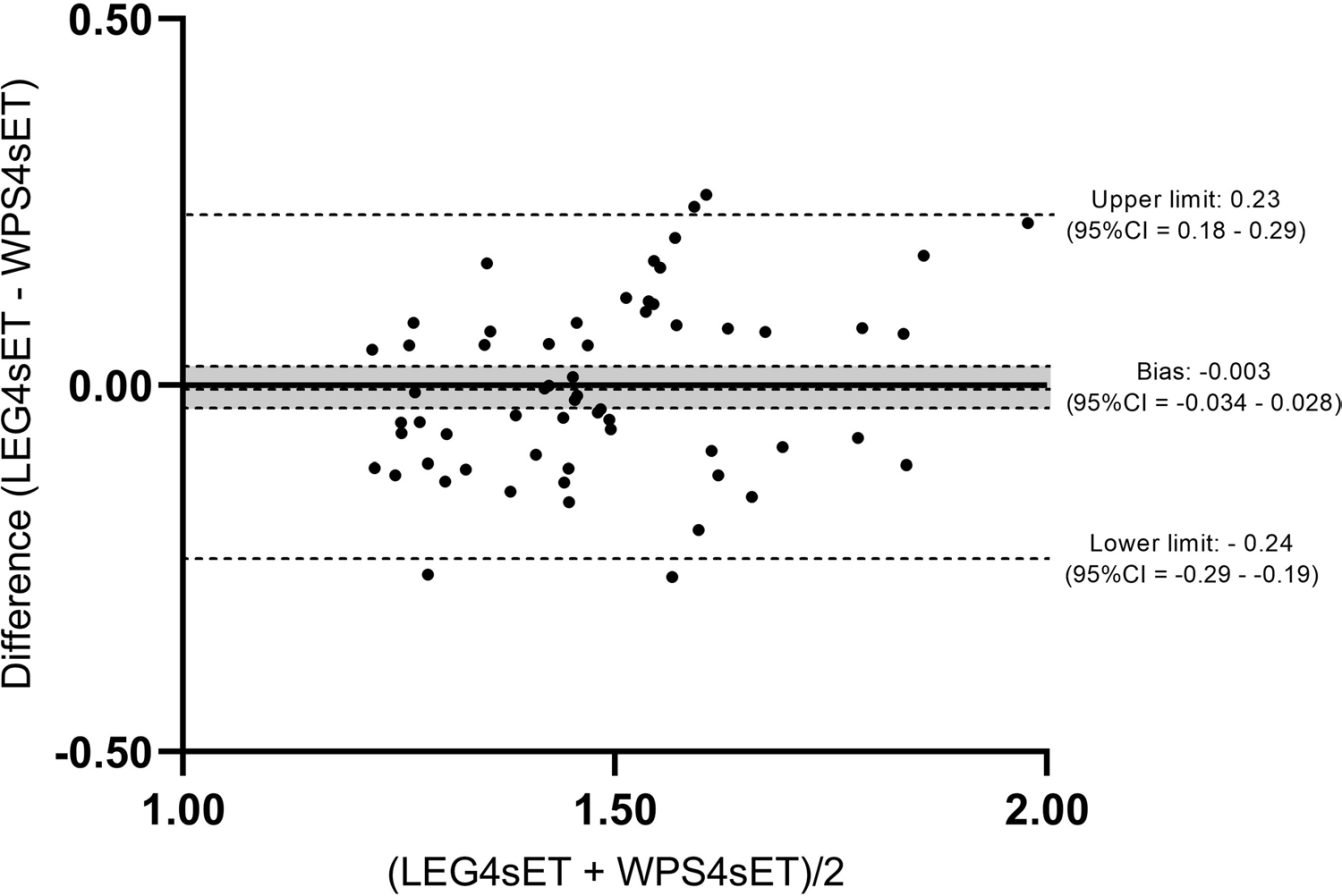
Bland–Altman plot assessing agreement between the cardiac vagal index (CVI) obtained from the LEG and WPS 4-s exercise test (4sET). The difference between measurements (LEG – WPS4sET CVI) is plotted against their mean. The solid horizontal line represents zero difference. The central dashed line indicates the mean bias, and the shaded area represents the 95% confidence interval of the bias. The upper and lower dotted lines denote the 95% limits of agreement (mean bias ± 1.96 × SD), indicating the range within which 95% of the differences between methods are expected to lie

Applying this correction eliminated the statistical difference between protocols (corrected WPS: 1.48 ± 0.03 vs. LEG: 1.48 ± 0.02; TEM = 5.8%; P = 0.867) while maintaining very good reliability (ri = 0.882; 95% CI = 0.803–0.930; P < 0.05), supporting the effectiveness of the proposed adjustment. Figure 3 shows Bland–Altman analysis of the corrected WPS, revealing a bias of -0.003 with 95% limits of agreement ranging from -0.241 to 0.235 . The mean difference between corrected WPS and LEG 4sET CVI values did not differ from zero (t =  − 0.19; P = 0.90). Moreover, the upper maximum allowed difference was larger than the limit of agreement, and the lower maximum allowed difference was lower than the lower limit of agreement for both protocols. As such, the level of disagreement between the 2 protocols was considered acceptable. Finally, no sex differences were observed in CVI (women: 1.46 ± 0.36 vs. men: 1.45 ± 0.36; P = 0.876), RRB (women: 949 ± 28 ms vs. men: 962 ± 28 ms; P = 0.741), or RRC (women: 657 ± 16 ms vs. men: 668 ± 16 ms; P = 0.625) across protocols.

## Discussion

This study compared cardiac vagal withdrawal at the onset of dynamic exercise using two approaches to the 4-s exercise test (4sET), a pharmacologically validated (Araújo et al. 1992) and highly reliable procedure (Araújo et al. 2003) that has been widely used over the past three decades to assess cardiac parasympathetic activity (Millar et al. 2009; Vianna et al. 2010; Teixeira et al. 2015; Ferreira et al. 2018, 2020; Oliveira et al. 2019; De Souza E Silva et al. 2022; Cuevas et al. 2023). The main finding is that wheelchair propulsion simulation (WPS) constitutes a valid movement to induce cardiac vagal inhibition during the first 4 s of exercise, as assessed by the WPS 4sET. Although initial CVI values were slightly lower in the WPS compared with the traditional 4sET performed with the lower limbs on an unloaded cycle ergometer, this difference was eliminated after applying the proposed regression correction for WPS CVI values below 1.50. Importantly, reliability between protocols, as indicated by ICC, was already very good before correction and remained essentially unchanged afterward (ri = 0.882; 95% CI = 0.803–0.930; P < 0.05). In addition, Bland–Altman analysis of the corrected data demonstrated a negligible bias and acceptable limits of agreement, confirming that the magnitude of disagreement between protocols was within clinically and physiologically acceptable ranges. These results support our hypothesis that WPS 4sET elicits cardiac vagal withdrawal comparable to the originally proposed unloaded cycle ergometer 4sET.

The differences initially observed in CVI between WPS and the traditional unloaded cycle ergometer 4sET may be explained by the relative contribution of peripheral afferents at exercise onset. Nobrega and Araújo (1993) demonstrated that the rapid HR increase at the onset of leg exercise is not dependent on volitional effort, but rather mediated by mechanically sensitive group III afferents (i.e., mechanoreflex). This was later confirmed by Teixeira et al. (2018), who reported similar chronotropic responses during the onset of both passive and active cycling. Although the magnitude of CVI itself does not depend on whether the effort is voluntary (Nobrega and Araújo 1993), the degree of mechanoreflex activation appears to influence the values obtained. Vianna et al. (2010) further showed that the amount of muscle mass involved in passive movements modulates RR interval duration at exercise onset, likely via progressive activation of stretch-sensitive mechanoreceptors. Because CVI is determined by the longest pre-exercise RR interval (RRB) and the shortest during exercise (RRC), differences in muscle mass recruited could explain the slightly lower CVI observed in the WPS compared with the leg protocol. Importantly, although Silva et al. (2008) reported similar vagal withdrawal between arm and leg exercise using a cycle ergometer, we hypothesize that the absence of handgrip engagement during WPS reduced mechanoreceptor feedback, contributing to the initial underestimation of CVI relative to the traditional protocol. Altogether, these physiological considerations help to explain the initial discrepancies between protocols and reinforce the need for the correction equation we propose to optimize comparability.

Considering previous evidence of sex-related differences in autonomic regulation, we also examined whether cardiac vagal withdrawal during the 4sET varied between men and women. Although some studies suggest that women may exhibit relatively stronger vagal influences on cardiac control and men a comparatively higher sympathetic drive (Koenig and Thayer 2016), our results showed no sex differences in the studied variables, as young men and women displayed similar RR interval durations and CVI values in both the LEG 4sET and the WPS 4sET protocols. Consistently, Matsuo et al. (2003) and Teixeira et al. (2018) reported no sex influence on HR responses at the onset of voluntary exercise or passive movement. Likewise, Teixeira et al. (2015) found that CVI is not affected by menstrual cycle phase or oral contraceptive use in physically active women. Araújo et al. (2015), analyzing 1,605 healthy adults tested with the 4sET between 1994 and 2014, also concluded that sex does not need to be considered in CVI interpretation. Altogether, these findings reinforce that cardiac vagal withdrawal at the onset of exercise is not influenced by sex in healthy adults, supporting the generalizability of our results across men and women.

While our study provides novel insights, some methodological considerations warrant attention. Our sample consisted exclusively of young adults between 18 and 28 years of age. Araújo et al. (2015), using the 4sET in a large cohort of 1,605 healthy adults, demonstrated that age-related declines in vagal function are gradual and tend to begin only after 30 years in both sexes, suggesting that our data reflect the stability of vagal control in early adulthood. Nevertheless, other studies using heart rate variability (HRV) indices have shown greater parasympathetic activity in women, potentially related to estrogen-mediated modulation within the central autonomic network (Abhishekh et al. 2013). It is important to note, however, that these discrepancies arise from methodological differences, as 4sET and HRV capture distinct aspects of autonomic regulation. Supporting this view, Paiva et al. (2011) found no association between 4sET and HRV in healthy subjects, suggesting complementary rather than overlapping mechanisms. Regardless of the method employed, substantial evidence indicates that aging reduces vagal activity (De Meersman and Stein 2007). Altogether, the lack of older participants in our sample may have limited the generalizability of the findings, particularly regarding the impact of aging on cardiac vagal withdrawal. Nevertheless, the availability of normative reference values for the 4sET (Araújo et al. 2015) enhances its applicability in diverse populations and provides a valuable framework for interpreting individual responses in clinical and research settings.

Our findings also provide a practical perspective, as there is currently no consensus on the best method to evaluate heart rate responses at the onset of exercise (Hettinga et al. 2014). Importantly, unlike widely usedHRV indices, which primarily reflect tonic autonomic activity under resting or steady-state conditions, CVI derived from the 4-s exercise test captures the rapid parasympathetic withdrawal that occurs during a distinct and physiologically relevant moment: the transition from rest to exercise. This distinction is supported by previous evidence demonstrating a low association between CVI and conventional HRV measures, indicating that these autonomic indices reflect complementary, rather than redundant, aspects of cardiac autonomic regulation (Paiva et al. 2011). In this context, the WPS 4sET emerges as a simple, low-cost, and easily executed alternative that may be particularly useful not only for individuals with motor impairments and wheelchair users, but also for clinical and research settings where a cycle ergometer is not available. This may be particularly relevant for individuals with spinal cord injury, who present increased cardiovascular risk and profound autonomic dysregulation (Soriano et al. 2025). In this population, exaggerated or unpredictable cardiovascular responses may occur even in response to minimal stimuli (Phillips and Krassioukov 2015), limiting the feasibility of conventional exercise-based assessments. In this context, a brief and low-load screening tool such as the WPS 4sET may help support clinical monitoring and longitudinal follow-up of autonomic function. In addition, the WPS 4sET may prove useful in rapid screening contexts, such as primary care, rehabilitation programs, or adapted sports settings, further reinforcing its translational potential. By relying on a movement that is intuitive and requires minimal equipment, the WPS 4sET broadens the applicability of cardiac vagal withdrawal assessment beyond the traditional laboratory environment.

In summary, our results indicate that the CVI obtained during the WPS 4sET reliably reflects cardiac vagal withdrawal at the onset of exercise. Importantly, WPS CVI values below 1.50 should be corrected using the proposed regression equation to optimize comparability and clinical applicability. Taken together, these findings establish the WPS 4sET as a valid, non-invasive, and safe tool to assess vagal autonomic function in a practical and inexpensive manner, with significant potential to support clinical decision-making and to be implemented in rapid screening environments, such as primary care, rehabilitation, and adapted sport programs.

## Data Availability

Data will be made available upon request.

## Abbreviation List

4sET: 4-Second exercise test
ANOVA: Analysis of variance
CI: Confidence interval
CVI: Cardiac vagal index
HR: Heart rate
HRV: Heart rate variability
ICC: Intraclass correlation coefficient
LEG 4sET: Leg cycle ergometer 4-s exercise test
RRC: Shortest RR interval duration during exercise
RRB: RR interval duration obtained immediately before the onset of exercise
SD: Standard deviation
SEE: Standard error of estimate
SEM: Standard error of the mean
TEM: Typical error of measurement
WPS 4sET: Wheelchair propulsion simulation 4-s exercise test
